# Examining gender differentials and determinants of private health insurance coverage in Zambia

**DOI:** 10.1186/s12913-021-07253-y

**Published:** 2021-11-09

**Authors:** James Mulenga, Mulenga C. Mulenga, Katongo M. C. Musonda, Chilizani Phiri

**Affiliations:** 1 Department of Economics, School of Social Science, Mulungushi University, Kabwe, Zambia; 2 National Authorizing Office of the European Development Fund, Ministry of Finance, Lusaka, Zambia; 3 Department of Economics, Uludag University, Bursa, Turkey

**Keywords:** Health insurance, Coverage, Gender, ZDHS, Zambia

## Abstract

**Background:**

Health insurance is an essential aspect of healthcare. This is because it enables the insured to acquire timely and essential healthcare services, besides offering financial protection from catastrophic treatment costs. This paper seeks to establish gender differentials and determinants of health insurance coverage in Zambia.

**Methods:**

The data used in this study was obtained from the 2018 Zambia Demographic and Health Survey. Data were analyzed using STATA 13.0 software and focused on descriptive and Probit regression analyses.

**Results:**

The study reveals that for women and men, age, wealth category, education, and professional occupation are positively associated with health insurance while being self-employed in the agricultural sector negatively influences health insurance coverage for both sexes. Other variables have gender-specific effects. For instance, being in marital union and having a clerical occupation increases the probability of having health insurance for women while being in the services, skilled, and unskilled manual occupations increases the probability of having health insurance for men. Further, residing in rural areas reduces the probability of having health insurance for men.

**Conclusion:**

The study concludes that there are differences in factors that influence health insurance between women and men. Hence, this study highlights the need to enhance health insurance coverage by addressing the different factors that influence health insurance coverage among men and women. These factors include enhancing education, job creation, diversifying insurance schemes, and gender consideration in the design of National Health Insurance Scheme.

## Background

Health insurance is one of the mechanisms of financing healthcare systems in many countries. Health insurance pools risks and ensures that the insured are protected financially against unexpected catastrophic health treatment costs, which may arise from unpredictable illness or injury [1]. Thus, risk and uncertainty regarding the timing and cost of treatment compel individuals to sign-up for health insurance schemes. Membership in such health insurance schemes brings forth various advantages to the insured and society at large. Through health insurance, individuals can get financial protection and avoid being thrown into dire financial vulnerability and poverty in times of illness or injury, through health insurance [2]. Health insurance thus enables insured families to effectively manage their savings, which could be wiped out in case of sudden illness or injury. It also enables the insured to have access to timely and quality healthcare services which can help achieve Universal Health Coverage (UHC).

Notwithstanding its importance to the insured and society, health insurance coverage is generally low in most developing countries [3]. Moreover, health insurance may not be equally distributed among men and women in a particular country. Women face greater challenges in the market for health insurance and are more likely to have higher direct healthcare expenses in comparison to men [4]. This poses a challenge to the realization of the Sustainable Development Goal (SDG) number 3, aimed at “ensuring healthy lives and promoting wellbeing for all at all ages” and in particular target 3.8, which focuses on “achieving universal health coverage, including financial risk protection, access to quality essential health-care services and access to safe, effective, quality and affordable essential medicines and vaccines for all” [5]. Adequate provision of healthcare, as well as healthcare financing systems that ensure access to adequate care regardless of ability to pay, is significant to achieve target 3.8. Achieving this target requires strengthening health systems, as well as having a robust financing structure and reducing out-of-pocket expenditure. It is a widely acknowledged fact that an efficient health financing system is critical to the achievement of UHC [6, 7].

Various studies have attributed healthcare utilization and health-seeking behavior to health insurance coverage [3, 8–10]. A study in Tanzania by Kibusi, Sunguya, Kimunai, et al. [11] established that having health insurance enhanced antenatal care visits. Another study undertaken in Ghana contended that being insured increased the probability of health facility deliveries [12]. In Kenya, Were, et al. [13] observed that health insurance enhanced the utilization of obstetric health services among HIV positive pregnant women. It has further been established that health insurance increases the use of private health services and lowers out-of-pocket payments [14]. These studies provide a link between health insurance and healthcare access as well as highlighting the importance of health insurance in the drive towards UHC.

Globally, three common types of health insurance schemes can be identified: Social Health Insurance (SHI), Community Based Health Insurance (CBHI), and Private Health Insurance (PHI). These different schemes vary in their requirements and coverage. SHI is a mandatory scheme in which individuals are compelled by law to enrol and pay a legally specified amount of premium. The benefits that accrue to those who are insured under this scheme are also determined by law. Many SHI schemes operate according to the solidarity principle where people contribute based on their ability to pay but enjoy the benefits according to needs. CBHI schemes are highly diverse  and defy efforts to arrive at a single definition. However, CBHI schemes are usually based on community membership and cover individuals who are excluded from other forms of health insurance. Premiums under CBHI can be paid in cash or kind.    The PHI is generally voluntary but may be compulsory too. Under this scheme, premiums are calculated based on the risks of the buyers of health insurance and may vary from insurer to insurer. PHI is a common type of health insurance scheme which plays an important role in most countries as a source of coverage for the population, complementing and supplementing the SHI on services not covered [15]. The scheme lacks the solidarity principle because it targets the well-to-do [16]. PHI is usually characterized by adverse selection, cream-skimming, and moral hazard problems.

Private health insurance (PHI) plays a critical role in health financing. It provides health insurance coverage for people who are not covered by social health insurance (SHI). In addition, it plays supplementary and complementary roles to public health insurance schemes. PHI can supplement SHIs by providing alternative coverage for healthcare institutions and healthcare services excluded from SHIs arrangements [17]. PHI can also compliment SHIs by providing double cover to individuals on SHI schemes. However, the existence of PHI is threatened by SHIs. A study by Jin, Hou, and Zhang [18] contends that public health insurance can push out private insurance. Another study by Zikusooka and Kyomuhangi [17] warned that SHI can negatively affect private health insurance schemes because employers shift to mandatory SHIs. To ensure an effective healthcare financing system (resource mobilisation and risk pooling) to achieve universal health insurance (UHC), there is a need to encourage the co-existence of SHI with the PHI, with PHI supplementing and complementing the SHI.

### Health financing in Zambia

**Fig. 1:**
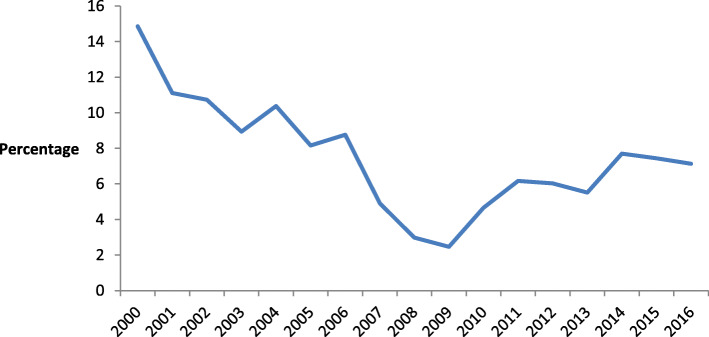
**Trends in domestic general government health expenditure as a percentage of general government expenditure in Zambia.** Source: Constructed using the World Development Indicators [6]

Before the implementation of the National Health Insurance Scheme (NHIS) in Zambia, financing of the health sector was mainly from general taxes, donor support, out-of-pocket payments, and PHI [19]. Total health expenditure as a percentage of total government expenditure in Zambia is low, as is the case in many developing countries. Zambia has not met the target set by the Abuja Declaration of having 15% of government annual expenditure allocated to the health sector. In recent years, government expenditure towards the health sector has consistently been below 15% of the general government expenditure. Figure 1 depicts government expenditure towards the health sector for the period 2000 to 2016. Zambia’s health expenditure is mainly dominated by external funding (42%), followed by general taxes (39%) and out-of-pocket payments (13%) [6].

**Fig. 2:**
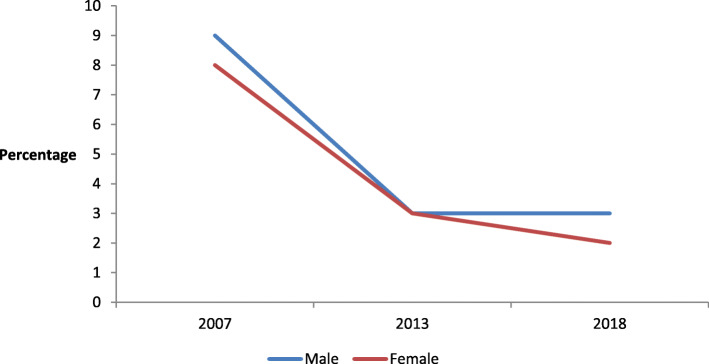
**Trends in health insurance coverage for women and men in Zambia.** Source: Constructed from [20–22]

Over the past years, the contribution of health insurance has generally been very low in Zambia. PHI has been very small, fragmented, and generally employer-based (low-cost prepayment scheme), although other schemes like social security and community-based schemes also exist [20, 21]. Figure 2 shows trends in health insurance for men and women in Zambia. The figure shows that, generally, health insurance coverage varies by sex in Zambia. Further, the figure shows a sharp decline in health insurance coverage among both sexes from 2007 to 2013. Between 2013 and 2018, health insurance coverage remained the same for men but declined slightly among women.  Hence, health insurance coverage was generally dominated by men between 2007 and 2018, despite insurance coverage being low among both sexes. For instance, in 2018, 3% of men were insured while only 2% of women had health insurance coverage. This represents a sharp decline in coverage, from 8 and 9% among women and men, respectively reported in the 2007 Zambia Demographic and Health Survey (ZDHS). Low rates of health insurance coverage imply that most Zambian citizens have to make direct out-of-pocket payments when they seek healthcare services or be catered for under government taxes. This makes them vulnerable to catastrophic health treatment costs [23].

The government of Zambia implemented the NHIS in 2019, to enhance the contribution of health insurance to national health financing. The NHIS is based on the solidarity model [24], where the risk is redistributed from healthy individuals to sick ones, from the rich to the poor, from the young to the elderly, and from small families to large families. The NHIS scheme is aimed at ensuring sustainable, predictable, and dedicated financing for the health sector and financial risk cover for Zambians [25].

The introduction of the mandatory NHIS poses a threat to the existence of private health insurance which may ultimately impact the progress towards the achievement of UHC. It is for this reason that this study was undertaken. This paper aims at establishing the gap in determinants of health insurance among men and women in Zambia. The study thus provides direction to private health insurance providers and policymakers on the determinants of health insurance between men and women in Zambia. It is envisaged that the output of this study will be used to encourage the growth of private health insurance to supplement and complement the NHIS, and help accelerate the pace towards UHC.

### Study objectives

The study aims at analyzing gender differentials and determinants of health insurance coverage in Zambia. Specifically, the study intends to achieve the following objectives:

1. To establish the difference in levels of health insurance coverage between women and men in Zambia
2. To compare factors influencing health insurance coverage between men and women in Zambia.

### Literature review

There is abundant scholarly work on factors affecting health insurance coverage in various countries around the world. However, few of these studies have focused on comparing the factors influencing health insurance coverage between women and men. The majority of these studies have focused on the determinants of health insurance coverage among women. In Zambia, studies comparing the factors influencing coverage to health insurance between women and men are non-existent. It is the aim of this study to fill up this knowledge gap.

A study by Wang and others [3] examined the levels of health insurance coverage among 30 low and middle-income countries. The research revealed that health insurance coverage was less than 5% in most of these countries except for eight (8) that had higher than 10%. The study further established that although the gender gap in health insurance coverage was generally narrow, it favoured men in 26 of the 30 countries studied. 

Due to patriarchal norms, traditions, and customs women are usually discriminated against and this leads to various gender inequalities in labour force participation rate, progression rate to higher education, access to health insurance among others. As a result of the discrimination and the fact men and women are confronted with varying factors, factors determining health insurance coverage may vary by sex too. Women have greater difficulty in signing up for health insurance due to various barriers. A study in China indicates that men are more likely to have health insurance from employers than women [26] due to lower labour force participation rates among women. This is confirmed by Dewar [27] who contended that the employment gender gap is a strong indicator for health insurance coverage because male-dominated industries have higher odds of health insurance coverage. Keene and Prokos [28] further showed that women with employed spouses had a lower probability of taking up health insurance.

Furthermore, related studies have identified various socioeconomic factors that have had a positive influence on health insurance. These socioeconomic factors include older age [29, 30], higher education level [3, 30–32], urban place of residence, wealth category [3, 21, 32], type of occupation [31], being married [30, 31], media exposure [30], household size, chronic disease, and knowledge of the benefits of and coverage of health insurance [33].

Although several studies [29, 30] show that, older age, being male, belonging to a rich household, and employment have positive effects on health insurance coverage, other studies [34–36] have contrasting results. For instance, studies by Duku [34] and Reshmi et al. [35] revealed that female-headed households have a higher likelihood of signing up for health insurance than male-headed households. Similarly, in Ghana, the people who lived in the households classified as poorest had higher national health insurance enrolments than those who lived in households classified as richest [36].

## Methods

### Data

This study utilized the latest Zambia Demographic and Health Survey (ZDHS) cross-sectional data which was collected under the sixth round of the ZDHS conducted between 18 July 2018 and 24 January 2019. The cross-sectional survey was executed by the Zambia Statistical Agency (ZamStats) in partnership with the Ministry of Health (MOH). The ZDHS survey based its sampling on the 2010 national census which was updated to accommodate the changes that occurred between 2010 and 2017.

A nationally representative sample of 12,831 households was selected for the survey using a two-stage stratified sample design. The sampling design included a selection of enumeration areas (EAs) and then a sample of households using systematic sampling. Twenty-five households were picked from each EA, with equal selection probability. According to International Coaching Federation [37], the two-stage stratified sample design was appropriate because it ensures representativeness of the sample, provides a sampling frame in cases where it is not available, enables the best coverage of the target population, and reduces sampling errors arising from more than two-stage sampling.

 The 2018 ZDHS  used four types of questionnaires to collect data namely: Household, Woman’s, Man’s, and Biomarker questionnaires. This study used data collected using the Woman's and Man's questionnaires.  The woman’s questionnaire was distributed to women aged 15 to 49 in sampled households, while the man’s questionnaire was used to collect data from men aged 15 to 59. To ensure that accurate data was collected, the questionnaires were translated into seven major languages. A pretest was also undertaken to ensure that there were no issues with the survey instruments before the full execution of the main survey.

The Institutional Review Board (IRB) of the ICF and the IRB in Zambia reviewed and approved all the questionnaires as well as Zambia’s specific survey protocols, to ensure adherence to ethical standards. The secondary data used in this study was obtained from the DHS Program website and was made available to the researchers on request.

## Data analysis

The Probit Regression Model was used to establish the factors that affect health insurance coverage. The model was estimated using the Maximum Likelihood method to generate marginal effects. The marginal effects show a change in the probability of the outcome variable when the independent variable changes by one unit [38]. This model was motivated by the fact that the dependent variable was binary or categorical. All the analyses were undertaken using STATA 13.0 software. The analysis was undertaken at a 95% confidence level. Sampling weights were used to adjust for oversampling in the ZDHS and avoid bias that would arise in the estimates, during data analysis. The study used pairwise correlation analysis to check for collinearity among the independent variables.

## Variables

Table 1 presents the variables used for data analysis. The variables were chosen based on existing empirical literature.

**Table 1:** Independent and Dependent Variables

DEPENDENT VARIABLE		
Variable Name	Description	Recode
Health Insurance coverage	Insured or not insured	Not insured = 0, insured = 1
**INDEPENDENT VARIABLES**		
**Variable Name**	**Description**	**Recode**
Age	Age of respondent in years (grouped)	15–24 = 0, 25–34 = 1, 35–44 = 2, 44 and above = 3
Marital Status	In marital union or not in marital union	Not in marital union = 0, in marital union = 1
Education	Education in single years	No recode
Occupation	Respondents type of occupation (grouped)	Not Working = 0, Professional = 1, Clerical = 2, Sales = 3, Sales = 4, Agricultural:Self-employed = 5, Household and domestic = 6, Services = 7, Skilled manual =8, Unskilled Manual = 9
Type of place of residence	Residing either in rural or urban	Urban = 0, rural = 1
Wealth Category	Household characteristics: Poor, medium, rich	Poor = 0, medium = 1, rich = 2
Media Exposure	Exposure to TV, Radio, Newspaper, and Magazines	Not exposed = 0, Exposed 1

## Study findings

### Characteristics of the sample

Characteristics of the sampled individuals for this study are presented in Table 2. Table 2 shows that 55.9% of the interviewed women are married while 53% of the men are married. The figure also shows that the majority of the interviewed women (41.9%) and men (39.7%) are in the age range of 15 to 24 years. In addition, 63.6% of the interviewed women and 79.8% of the interviewed men had exposure to some mass media. About 46.6% of the women are from households classified as rich, while 45.7% of the men are from rich households. The majority of the women have primary education (44.3%) while the majority of the men (48.8%) have secondary education. About 53.4% of the women and 55.9% of the men reside in rural areas. In terms of occupation, a higher percentage (47.9%) of the interviewed women reported not being employed, while the majority of the men (25.4%) are self-employed in the agricultural sector.

**Table 2:** Characteristics of the study sample: Women versus men

	FEMALE (%)	Sample (N)	MALE (%)	Sample (N)
**Marital Status**				
Not in marital union	44.1	6035	47	5704
In marital union	55.9	7648	53	6428
**Age Group**				
15–24	41.9	5733	39.7	4813
25–34	30	4100	25.6	3104
35–44	21.6	2950	19.6	2377
45+	6.6	900	15.2	1838
**Media Exposure**				
No Exposure	36.4	4979	20.2	2454
Exposed	63.6	8704	79.8	9678
**Wealth Category**				
Poor	35.3	4828	34.3	4164
Middle	18.1	2477	20	2422
Rich	46.6	6377	45.7	5546
**Educational level**				
No education	7.7	1054	4.1	492
Primary	44.3	6059	38.9	4722
Secondary	42.5	5816	48.8	5918
Tertiary	5.5	755	8.2	1000
**Type of place of residence**				
Urban	46.6	6374	44.1	5346
Rural	53.4	7309	55.9	6786
**Occupation Type**				
Not Working	47.9	6547	20.6	2493
Professional	4	550	7	843
Clerical	0.5	73	0.5	61
Sales	15.7	2146	6.5	785
Agriculture - self employed	17.9	2443	25.4	3074
Household and domestic	4.4	600	4.9	596
Services	2.4	330	3.6	432
Skilled manual	1	137	15.9	1927
Unskilled manual	6.2	844	15.8	1911

### Bivariate analysis of health insurance coverage among women and men in Zambia

Table 3 presents the percentage distribution of health insurance coverage in Zambia by sex and socioeconomic characteristics. The table indicates that among insured men 4% were in marital union while 2.1% were not in marital union. For insured women, 2.4% were in marital union while 1.7%. The table also shows that women and men who have the highest proportion (4.4 and 6.7% respectively) of health insurance belong to rich households. Women and men from poor households have no insurance coverage. The results also show that women and men with tertiary education have the highest proportion of health insurance coverage (21.8 and 24.5%, respectively) in comparison to those who have no education - primary and secondary education. The table further shows that health insurance coverage is highest among women and men in the clerical occupation (29.9 and 21.5%, respectively) and lowest among women and men in the agricultural sector (self-employed) with 0.1 and 0.2%, respectively.

**Table 3:** Percentage of men and women with health insurance according to demographic and socio-economic characteristics

	Men					Women				
Variable	Not Insured		Insured		*P*-value	Not Insured		Insured		P-value
	%	CI	%	CI		%	CI	%	CI	%
**Marital union**										
Not in union	97.9	[97.2,98.5]	2.1	[1.5,2.8]	0.0000	98.3	[97.7,98.8]	1.7	[1.2,2.3]	0.0000
In union	96	[95.0,96.8]	4	[3.2,5.0]		97.6	[96.9,98.2]	2.4	[1.8,3.1]	
**Age Group**										
15–24	98.9	[98.0,99.4]	1.1	[0.6,2.0]	0.0000	99	[98.5,99.4]	1	[0.6,1.5]	0.0000
25–34	96.4	[95.3,97.2]	3.6	[2.8,4.7]		97	[95.9,97.8]	3	[2.2,4.1]	
35–44	95.8	[94.3,96.8]	4.2	[3.2,5.7]		97.2	[96.2,97.9]	2.8	[2.1,3.8]	
45+	94.1	[92.2,95.5]	5.9	[4.5,7.8]		97.9	[96.1,98.9]	2.1	[1.1,3.9]	
**Media Exposure**										
No access/exposure	99.8	[99.5,99.9]	0.2	[0.1,0.5]	0.0000	99.8	[99.7,99.9]	0.2	[0.1,0.3]	0.0000
Access to media/exposed	96.2	[95.3,96.9]	3.8	[3.1,4.7]		96.9	[96.0,97.6]	3.1	[2.4,4.0]	
**Wealth Index**										
Poor	100	[99.9100.0]	0	[0.0,0.1]	0.0000	100	[99.9100.0]	0	[0.0,0.1]	0.0000
Middle	99.8	[99.5,99.9]	0.2	[0.1,0.5]		99.9	[99.6100.0]	0.1	[0.0,0.4]	
Rich	93.3	[91.9,94.5]	6.7	[5.5,8.1]		95.6	[94.4,96.6]	4.4	[3.4,5.6]	
**Education Level**										
No education	96.7	[87.4,99.2]	3.3	[0.8,12.6]	0.0000	100		0		0.0000
Primary	99.7	[99.3,99.9]	0.3	[0.1,0.7]		99.9	[99.8100.0]	0.1	[0.0,0.2]	
Secondary	98.3	[97.8,98.7]	1.7	[1.3,2.2]		98.1	[97.2,98.7]	1.9	[1.3,2.8]	
Tertiary	75.5	[71.1,79.4]	24.5	[20.6,28.9]		78.2	[73.0,82.7]	21.8	[17.3,27.0]	
**Type of place of residence**										
Urban	93.9	[92.4,95.1]	6.1	[4.9,7.6]	0.0000	96.1	[94.8,97.1]	3.9	[2.9,5.2]	0.0000
Rural	99.3	[98.9,99.5]	0.7	[0.5,1.1]		99.6	[99.3,99.7]	0.4	[0.3,0.7]	
**Occupation**										
Not working	99.4	[98.9,99.7]	0.6	[0.3,1.1]	0.0000	98.8	[98.3,99.2]	1.2	[0.8,1.7]	0.0000
Professional/technical/managerial	78.2	[73.7,82.1]	21.8	[17.9,26.3]		80.8	[75.8,84.9]	19.2	[15.1,24.2]	
Clerical	78.5	[58.5,90.5]	21.5	[9.5,41.5]		70.1	[54.8,82.0]	29.9	[18.0,45.2]	
Sales	98.3	[96.6,99.2]	1.7	[0.8,3.4]		98.6	[97.7,99.1]	1.4	[0.9,2.3]	
Agriculture:self-employed	99.8	[99.4100.0]	0.2	[0.0,0.6]		99.9	[99.4100.0]	0.1	[0.0,0.6]	
Household and domestic	99.2	[97.3,99.7]	0.8	[0.3,2.7]		98.6	[96.8,99.4]	1.4	[0.6,3.2]	
Services	92.8	[88.9,95.4]	7.2	[4.6,11.1]		95.5	[89.3,98.2]	4.5	[1.8,10.7]	
Skilled manual	96.2	[94.8,97.3]	3.8	[2.7,5.2]		93	[85.4,96.8]	7	[3.2,14.6]	
Unskilled manual	98	[96.3,98.9]	2	[1.1,3.7]		98.7	[97.5,99.3]	1.3	[0.7,2.5]	

### Determinants of health insurance Coverage among men and women

This section presents the results of the Probit regression analysis for determinants of health insurance coverage among women and men in Zambia. These results are presented in Table 4. Table 4 shows the Probit marginal effects and their corresponding probability values (*p*-values). The results show that being married, being in the 35 to 44 age category, a rich household, higher education, professional, clerical, or agriculture occupation are significantly associated with health insurance among women. For men; age, being from a rich household, higher education, place of residence, being in a profession, agriculture, services, or skilled manual occupations significantly influence health insurance.

**Table 4:** Probit Regression Analysis of the Factors Influencing Health Insurance Coverage: Women versus Men

	WOMEN		MEN	
VARIABLE	Marginal Effects (dy/dx)	P-values	Marginal Effects (dy/dx)	***P***-values
**Marital Status**				
Not in Marital Union	1			
In Marital Union	0.0115	0.000	0.0053	0.137
**Age Group**				
15–24	1			
25–34	0.0028	0.307	0.0091	0.019
35–44	0.0083	0.023	0.0099	0.033
45 and above	0.0096	0.114	0.0213	0.000
**Wealth Category**				
Poor	1			
Middle	0.0015	0.637	0.0042	0.254
Rich	0.0172	0.000	0.0263	0.000
**Education in single years**	0.0056	0.000	0.0055	0.000
**Type of Place of Residence**				
Urban	1			
Rural	−0.001	0.682	−0.0083	0.005
**Media Exposure**				
Not Exposed	1			
Exposed	0.0062	0.059	0.0074	0.238
**Occupation**				
Not in Employment	1			
Professionals	0.0095	0.024	0 .0320	0.000
Clerical	0.0387	0.007	0.0207	0.120
Sales	−0.0039	0.153	−0.0038	0.383
Agricultural - self employed	−0.011	0.003	− 0.0091	0.028
Household and domestic	0.0045	0.472	−0.0039	0.507
Services	0.0006	0.902	0.0208	0.004
Skilled manual	0.0226	0.080	0.0113	0.011
Unskilled manual	0.0051	0.324	0.0122	0.023

The results show that there is an increase in the probability of having health insurance (about 1.15%) among women in marital union compared to women that are not in marital union. Generally, the probability of having health insurance increases with age for both sexes. For women in the age category 35 to 44, the probability of having health insurance increases by 0.83% compared to women in the age group of 15 to 24. Men in higher age categories (25 to 34, 35 to 40, and above 40) are more likely to be insured compared to those aged between 15 and 24 years, holding other variables constant. The probability of having health insurance for men in the age categories of 25 to 34, 35 to 44, and above 45 years is 0.91, 0.99, and 2.13% higher than men aged between 15 and 24 years, respectively.

The results also show that men and women from households classified as rich have a higher probability of having health insurance (1.72% for women and 2.63% for men) compared to those from households classified as poor. Further, as the years of education increase by 1, the probability of having health insurance increases by 0.56% for women and 0.55% for men. The results show that men living in rural areas have a lower probability (0.83%) of health insurance relative to their urban counterparts. Being in professional and clerical occupations enhances the probability of being insured by 0.95 and 3.87%, respectively for women. However, being self-employed in the agricultural sector reduces the probability of having health insurance for women by 1.1%. For men in the professional, services, skilled manual and unskilled manual, the probabilities increase by 3.20, 2.08, 1.13, and 1.22%, respectively. Being self-employed in the agricultural sector reduces the probability of men having health insurance by 0.91%, similar to women.

## Discussion of findings

This study has established that private health insurance coverage in Zambia has generally been low for both women and men. Similar patterns have been observed by other studies in other developing countries. The results also show that health insurance coverage in Zambia varies with sex, with coverage favouring the male. The results of this study are in tandem with the findings of Wang et al. [3] who observed that health insurance coverage in most developing countries was less than 5% and that the gender gap in health insurance coverage favored men. The study further shows that education, wealth, or occupation do not reduce the health insurance gender gaps.

Further analysis of data using the Probit regression model provides factors that significantly influence the health insurance coverage of women and men in Zambia. According to the results, being in a marital union was found to increase the probability of signing up for health insurance in comparison to not being in a marital union for both sexes. This is possible because people in marital unions can pool resources together and be able to afford health insurance. The finding is consistent with [31] who contended that people who are married have higher a possibility of having health insurance than those who are not married in Ghana. Similar results were observed by Kazungu and Barasa [39] in Kenya and Mulenga et al. [30] in Zambia.

The age of the respondent is also another important factor that increases the probability of signing up for health insurance. It is believed that as individuals age, their health deteriorates at a faster rate than younger individuals. It is for this reason that the elderly are more likely to sign up for health insurance to cover themselves against the heightened risk of illness [40]. These findings are consistent with the findings of other studies [29, 31, 39] undertaken in other countries.

The results of this study further show that men and women from households classified as rich have a higher probability of having health insurance compared to those who live in households classified as poor. A study by Wang et al. [3] in 30 low and middle-income countries and another study by Amu et al. [29] in Ghana, Kenya, Nigeria, and Tanzania had similar findings.

The number of years in education was found to increase the probability of health insurance coverage for both women and men. Education plays an important role in people’s lives as it enables individuals to understand their health needs and make informed choices. Education can also enhance healthcare information-seeking behavior and quality decision-making [41]. Education further enables individuals to get well-paying jobs which give them a higher ability to purchase health insurance.

In terms of place of residence, the results show that men who reside in rural areas have a lower probability of having healthcare insurance. This can be attributed to the high levels of poverty among the majority of rural dwellers. In Zambia, 54.4% of the people live below the national poverty line with 76.6% in rural areas [42]. People in rural areas are also generally not well educated. Moreover, private insurance companies are located in urban areas [30].

The type of occupation one engages in can greatly influence the demand for and coverage of health insurance. The results indicate that some occupations are associated with an increase in the probability of signing up for health insurance while others decrease the probability of health insurance coverage. It has been established on one hand that, being in professional and clerical occupations increases the probability of signing up for health insurance for both women and men. On the other hand, being self-employed in the agricultural sector reduces the probability of having health insurance for both sexes.

The results of the study further show that men in services, skilled manual, and unskilled manual occupations had an increased probability of having health insurance coverage. Most of these occupations such as professional, clerical, and skilled manual are found in the formal sector characterized by paid jobs. As a result, income generated from such jobs gives an individual greater ability to purchase health insurance. Moreover, in most formal jobs health insurance comes as part of the employment package [43]. In this case, employers fund their employees’ medical expenses directly [44] or through a joint employer-employee contributions to a scheme. The agricultural sector is generally a rural-based and most people engaged in the agricultural sector are usually subsistence self-employed farmers who do not generate enough revenue to afford health insurance.

### Policy implications and study limitations

Based on the results presented, the following are the policy implications of the findings of this study:

The Ministry of General Education must enhance and encourage primary education for all. This is because enhanced education enlightens individuals and enables them to make informed choices. As such, enhancing education can boost health insurance coverage. Hence, enhancing access to education would boost health insurance coverage for both men and women in Zambia, since education is positively associated with health insurance.

Introduction of community health insurance schemes to cover men and women in agriculture. Specifically, those who may not have the cash to pay premiums under the SHI or private health insurance but may have the capacity to pay in kind.

Enhancing employment creation, particularly formal employment, can boost health insurance coverage. This is because cash payments give individuals the ability to pay premiums for health insurance.

Government should consider the findings of this study, taking note of gender differentials, when designing policies and strategies under the NHIS. Thus the design of health insurance policies should not be gender blind.

It is worth noting some limitations of this study. The variables used for analysis in this study are limited to the variables found in the 2018 ZDHS. Several key explanatory variables such as premiums, benefits covered under health insurance, quality of healthcare, etc. are not available in the ZDHS. Omission of such important variables from the model may lead to specification bias. In place of premiums, the wealth index acts as a proxy. Thus future research should focus on capturing the important variables.

## Conclusion

The study has observed a drop in health insurance rates in recent years in Zambia. Currently, health insurance coverage is generally very low, although coverage tends to favour the male. The study has also established that there are variations in the factors affecting health insurance coverage among women and men, although they share common factors too. For both men and women; age, wealth category, education, and professional occupation are positively associated with health insurance while being self-employed in the agricultural sector is negatively associated with health insurance coverage. It has been further established that marital union and clerical occupation only increase the probability of signing up for health insurance for women. In the same vein, residing in the rural areas, being in the services, skilled manual and unskilled manual only increases the probability of signing up for health insurance for women. Similarly, residing in rural areas reduces the probability of health insurance. Thus, all these factors point to the fact that there are differentials in the factors that influence health insurance coverage.

## Data Availability

The secondary data used in this study was obtained from the DHS Program website and was made available to the researchers on request. Data for this study is available at: https://dhsprogram.com/data/dataset/Zambia_Standard-DHS_2018.cfm?flag=0
